# Nanoporous Graphene Integrated onto Bimodal Waveguide Biosensors for Detection of C-Reactive Protein

**DOI:** 10.1021/acsanm.4c06716

**Published:** 2025-01-10

**Authors:** Bárbara Lisboa, Maria Soler, Rukmani Singh, Jesús Castro-Esteban, Diego Peña, Aitor Mugarza, Laura M. Lechuga, César Moreno

**Affiliations:** † Nanobiosensors and Bioanalytical Applications Group (NanoB2A), Catalan Institute of Nanoscience and Nanotechnology (ICN2), CSIC, BIST and CIBER-BBN, Bellaterra, 08193 Barcelona, Spain; ‡ Atomic Manipulation and Spectroscopy Group (AMS), Catalan Institute of Nanoscience and Nanotechnology (ICN2), CSIC and BIST, Bellaterra, 08193 Barcelona, Spain; § Centro de Investigación en Química Biolóxica e Materiais Moleculares (CiQUS) and Departamento de Química Orgánica, Universidade de Santiago de Compostela, 15782 Santiago de Compostela, Spain; ∥ Oportunius, Galician Innovation Agency (GAIN), 15702 Santiago de Compostela, Spain; ⊥ ICREA − Institució Catalana de Recerca i Estudis Avançats, 08010 Barcelona, Spain; # Departamento de Ciencias de la Tierra y Física de la Materia Condensada, Universidad de Cantabria, 39005 Santander, Spain

**Keywords:** 2D materials, on-surface synthesis, nanoporous graphene, biofunctionalization, interferometric waveguide, photonic biosensor, diagnosis

## Abstract

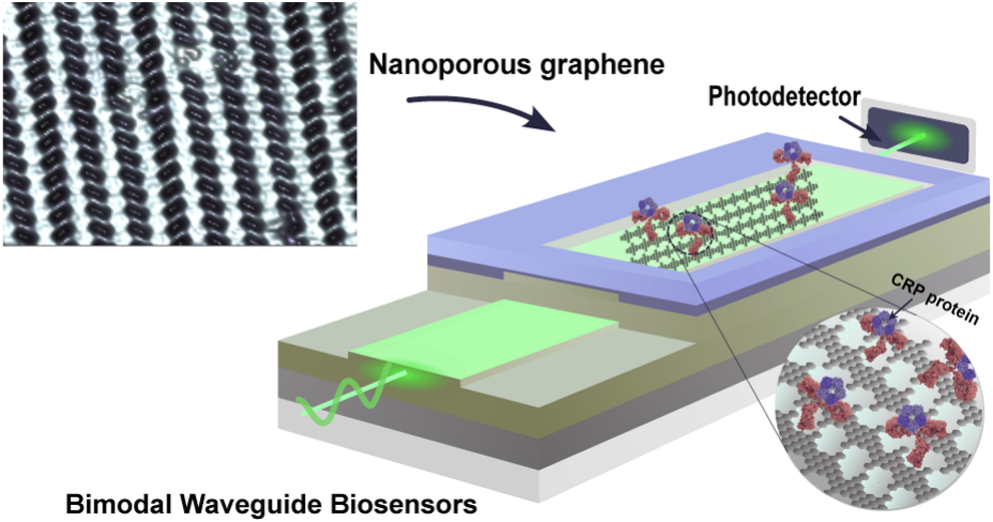

Despite the outstanding progress in photonic sensor devices, a major limitation for its application as label-free biosensors for biomedical analysis lies in the surface biofunctionalization step, that is, the reliable immobilization of the biorecognition element onto the sensor surface. Here, we report the integration of bottom-up synthesized nanoporous graphene onto bimodal waveguide interferometric biosensors as an atomically precise biofunctionalization scaffold. This combination leverages the high sensitivity of bimodal waveguide interferometers and the large functional surface area of nanoporous graphene to create highly sensitive, selective, and robust biosensors for the direct immunoassay detection of C-reactive protein (CRP), an inflammatory biomarker widely used in the clinical diagnosis of infections and sepsis. The limit of detection was determined at 3 ng/mL, which is well below the clinical cutoff levels required for the diagnostic detection of CRP in patient samples. This innovative approach holds promise for transforming diagnostics, environmental monitoring, and various fields requiring precise biomolecular detection.

## Introduction

Nanoporous graphene (NPG) is a two-dimensional (2D) nanostructured material characterized by a high surface-to-volume ratio, chemical stability, and a high density and periodic arrangement of reactive pores.^1^ The combination of these properties in a single atomically thin and mechanically robust sheet makes NPG an ideal platform for its integration as a biofunctionalization scaffold in biosensors. Presynthesized functional NPG sheets could serve as a precise template for the efficient immobilization of bioreceptors with a controlled distribution and grafting density. However, the exploitation of the maximum potential of this material depends on the ability to create atomically precise periodic nanopores. To date, top-down methods have been inadequate because they typically produce randomly located pores with varied sizes.^2^ Conversely, bottom-up methods provide atomic precision in NPG, which can be tailored with specific pore sizes and functionalities through the selection of suitable molecular building blocks.^1,3−6^ Naturally, NPG has a high density of hydrogenated bonds at the chemically reactive pore edges, offering active sites for chemical postmodification.^7^ In addition, various strategies have been developed over the past few years to create graphene nanoribbons containing functional chemical groups.^5,8−12^ Although only a few of the on-surface synthesized graphene nanoribbons have led to nanoporous graphene,^1,3,4,13,14^ it is expected that soon the variety of nanoporous graphene with functionalized groups will develop largely driven by the rise of the on-surface synthesis field. The achieved accurate control over the structure and composition of bottom-up synthesized NPG material could provide a functional, atomically precise, and versatile interface that could simplify and improve the sensor biofunctionalization procedures.^15^ Recently, the use of solution-based graphene nanoribbons has recently been published as a versatile strategy for covalent anchoring of bioreceptors, allowing selective and sensitive detection of analytes.^16^ This alternative approach could represent significant progress in the development of biofunctionalization procedures with respect, for instance, to those based on silanization approaches,^17^ which are still quite complex and laborious, requiring a wide range of parameters to be controlled and optimized. Suboptimal optimization of these processes could lead to low sensitivity, selectivity, and reproducibility of the final biosensor device.

In this work, we develop the unexplored integration of bottom-up synthesized NPG as a biofunctionalization scaffold for silicon photonics biosensors. We have applied a functional NPG template as a biofunctionalization interface for one of the most sensitive and widely demonstrated silicon photonics biosensors, the bimodal waveguide (BiMW) interferometer, a device invented and fully developed in our group.^18−20^ This biosensor operates on the principle of light confinement and propagation along a straight optical waveguide made of silicon nitride (Si_3_N_4_), generating an evanescent field that is highly sensitive to refractive index (RI) changes occurring on the sensor surface, such as those caused by biomolecular interaction. In particular, the bimodal waveguides are designed to work within the visible range, allowing for the confined propagation of two light modes (the fundamental and the first-order modes), whose respective evanescent fields behave differently to the RI changes happening in the sensing window. This results in different propagation wave parameters, causing an interferometric phase shift (ΔΦ) at the waveguide output that can be readily monitored. This sensing principle allows for the direct detection and quantification of specific analytes in a few minutes when the sensor surface is functionalized with the corresponding bioreceptor, reaching sensitivities in the 10^–9^ molar (nM) to 10^–15^ molar (fM) range in a label-free format and without any amplification.^21−26^ Besides, the BiMW sensors are fabricated through well-established cost-effective standard microelectronics production techniques at clean-room foundries, facilitating their integration in small-footprint devices with multiplexing capacity for the parallel analysis of different analytes,^27−31^ which is key for point-of-care (POC) testing and precision diagnostics. However, the BiMW surface biofunctionalization remains one of the major challenges for its technology transfer and clinical implementation, as happens to many other biosensors, being one of the bottlenecks for their ample commercialization. With our proposal of incorporating functional NPG into BiMW sensors, we intend to provide a solution to this problem. For that, we integrate a presynthesized template for precise and efficient anchoring of bioreceptors via chemical cross-linking to the activated pore regions. The novel NPG-BiMW device has been studied and optimized in terms of interferometric sensing performance and it has been demonstrated as a functional biosensor for the direct immunoassay detection of C-reactive protein (CRP), an inflammatory biomarker widely used in clinical diagnosis.

## Experimental Section

### Materials

Organic solvents (acetone, ethanol, methanol, and isopropanol) were purchased from Panreac (Barcelona, Spain). Reagents for the transfer, chlorhydric acid (HCl), potassium iodide/iodine solution (KI/I_2_); for sensor characterization, dimethyl sulfoxide (DMSO); for carboxylic acid activation, *N*-(3- dimethyl aminopropyl)-*N*′-ethyl carbodiimide hydrochloride (EDC) and *N*-hydroxysulfosuccinimide (sulfo-NHS); salts for phosphate buffered saline solution **(**PBS) 10 mM (10 mM PBS, 2.7 mM KCl, 137 mM NaCl, pH 7.4), and MES 0.1 M (2-(*N*-morpholino) ethanesulfonic acid, pH 5.5), ethanolamine (EA 1 M, pH 8), and bovine serum albumin (BSA) were provided by Sigma-Aldrich/Merck (Steinheim, Germany). Milli-Q water was employed for all the buffer preparation. Recombinant anti-CRP antibody (4C28, C6 cm^3^) was purchased from HyTest (Turku, Finland), and CRP protein from BBI solutions (Freiburg, Germany). For the microfluidics fabrication, Sylgard 184 PDMS and elastomer were acquired from Darwin Microfluidics (Spain). All biomolecules employed in this work were prepared in the same buffer by serial dilution from high-concentration stocks, ensuring identical sample matrix components.

### NPG Sample Preparations

Commercial gold thin films (300 nm thickness, Georg Albert PVD) on mica substrate (ca. 1.3 cm × 0.8 cm) were used to grow the NPG material. Gold substrates were introduced on an ultrahigh vacuum (UHV) chamber with a base pressure of 1 × 10^–9^ mbar. Substrates were prepared by repeated sputter–annealing cycles using Ar^+^ ions at an energy of 1 keV and annealing to ca. 470 °C. The synthesis of 10,10-dibromo 9,9-bianthracene (DP-DBBA) used as NPG molecular precursor, has been reported previously.^1^ Topographic measurements were carried out using a commercial Aarhus 150 variable temperature scanning tunneling microscope (STM) in constant current mode. Image processing was performed using WSxM software.^32^

### BiMW Sensor Chip

The BiMW is fabricated at wafer-scale at the ICTS cleanroom facility of the National Microelectronics Center (IMB-CNM-CSIC, Barcelona, Spain) as previously described.^18^ Each BiMW sensor microchip (3 cm × 1 cm) contains an array of 20 individual Si_3_N_4_ straight rib bimodal waveguides (3 μm width, rib of 1–3 nm and 250 μm pitch between waveguides), with a single-mode region (150 nm core thickness) where only the fundamental mode can propagate, followed by a step junction to excite the fundamental and first-order modes on the bimodal region (340 nm core thickness), a sensing window of 15 mm × 0.05 mm is opened at the bimodal region. Detailed description of the BiMW sensing mechanism, data acquisition, and data analysis is provided in the Supporting Information (Section A, SI). Specific coating of SiO_2_ on the sensing waveguides as a spacer layer and for reduction of the sensing area is done by electron beam deposition (AJA International Inc. ATC-8E, Orion). Before use, the BiMW chip is cleaned by sonication of different acetone, ethanol, and water cycles for 5 min, finishing with 1:1 (v/v) methanol/hydrochloric acid (MeOH/HCl) solution for 10 min at 60 °C, then rinsed with Milli-Q H_2_O and dried under an N_2_ stream.

### NPG Transfer onto the BiMW

The transfer of NPG onto the BiMW sensor is adapted from a previously reported procedure.^1^ Briefly, first, the NPG/gold film is detached from the mica substrate by immersing it in an HCl 37% bath solution for 3 h. Later, the sample is moved to a Milli-Q H_2_O bath solution and left floating. After fishing and adhering the NPG/Au film to the BiMW device, it is annealed on a hot plate at 150 °C for 20 min. Finally, a gold etchant KI/I_2_ solution (0.2/0.05 M) is applied for 1 h at 40 °C. The NPG-BiMW chip is rinsed and sonicated for 20 min with Milli-Q H_2_O and isopropanol and dried under nitrogen flow.

### RAMAN and XPS Characterization

Raman spectroscopy was performed by using a WITec Raman spectrometer, using a 532 nm excitation laser (*P* = 0.5 mW), with a 50× focal objective, 600 g/mm grating, 0.5 mW maximum power, and 0.05 s acquisition time. The Raman spectra were processed using WITec Project Five software. The oxidized-NPG samples were analyzed by an X-ray spectroscopy (XPS) SPECS PHOIBOS 150 hemispherical spectrometer (SPECS GmbH, Berlin, Germany), equipped with a monochromatic aluminum Kα radiation X-ray source at a base pressure of ca. 5 × 10^–10^ mbar, with a pass energy of 20 eV and a step size of 0.05 eV.

### Antibody Immobilization and CRP Protein Detection

Before antibody immobilization, NPG was functionalized by oxygen plasma treatment in a Femto equipment from Diener electronic (Aname, Spain). The plasma chamber was filled with O_2_ gas for 1 min at a pressure of 445 sccm, and NPG was exposed to 12 s of oxygen reaction followed by 1 h of annealing at 180 °C on a hot plate. The NPG-BiMW device is mounted on the optical setup to carry out the biosensing assay on the individual waveguides by employing the microfluidics platform. Carboxyl (COOH), epoxy (COC), carbonyl (CHO), and hydroxyl (OH) groups provided by the NPG layer are activated through EDC/NHS reaction (0.2 M EDC/0.05 M NHS in MES buffer 0.1 M, pH 5.5). Next, a solution with anti-CRP antibody (20 μg/mL in MES buffer) is injected at 20 μL/min. The remaining activated oxide groups are blocked with ethanolamine (1 M, pH 9) for 2 min. Milli-Q water was used as the running buffer during the immobilization step and was then changed to slightly diluted PBS buffer (0.9x PBS, pH 7.4) for CRP detection. CRP solutions were prepared in 1× PBS at different concentrations (0.05–1 μg/mL) and injected at 10 μL/min at increasing concentrations. The minimal dilution of the running buffer does not affect the biological activity of antibodies, and it enhances signal clarity by inducing a bulk refractive index change for the protein samples. For the biosurface regeneration, HCl (10 mM, pH 2) was injected for 1 min. All experiments were performed at a constant temperature of 23 °C.

### Biosensor Data Analysis

Data was analyzed using Origin Pro 2018 (OriginLab, MA) and GraphPad Prism 9 (GraphPad Software, CA). Calibration curves were plotted as the mean and standard deviation of the acquired sensor response (ΔΦ) over the analyte concentration. Data points were fitted to a linear regression model for bulk sensitivity evaluation, and to a one-site specific binding model curve for biosensing experiments. The LOD, defined as the smallest concentration distinguishable from the blank, was determined as the concentration corresponding to three times the standard deviation of the baseline for over 1000 data points. All sensor signals, including blank signals, were obtained by duplicate measurements with different biosensors.

## Results and Discussion

### NPG Synthesis and Transfer onto BiMW Sensors

**Figure 1:**
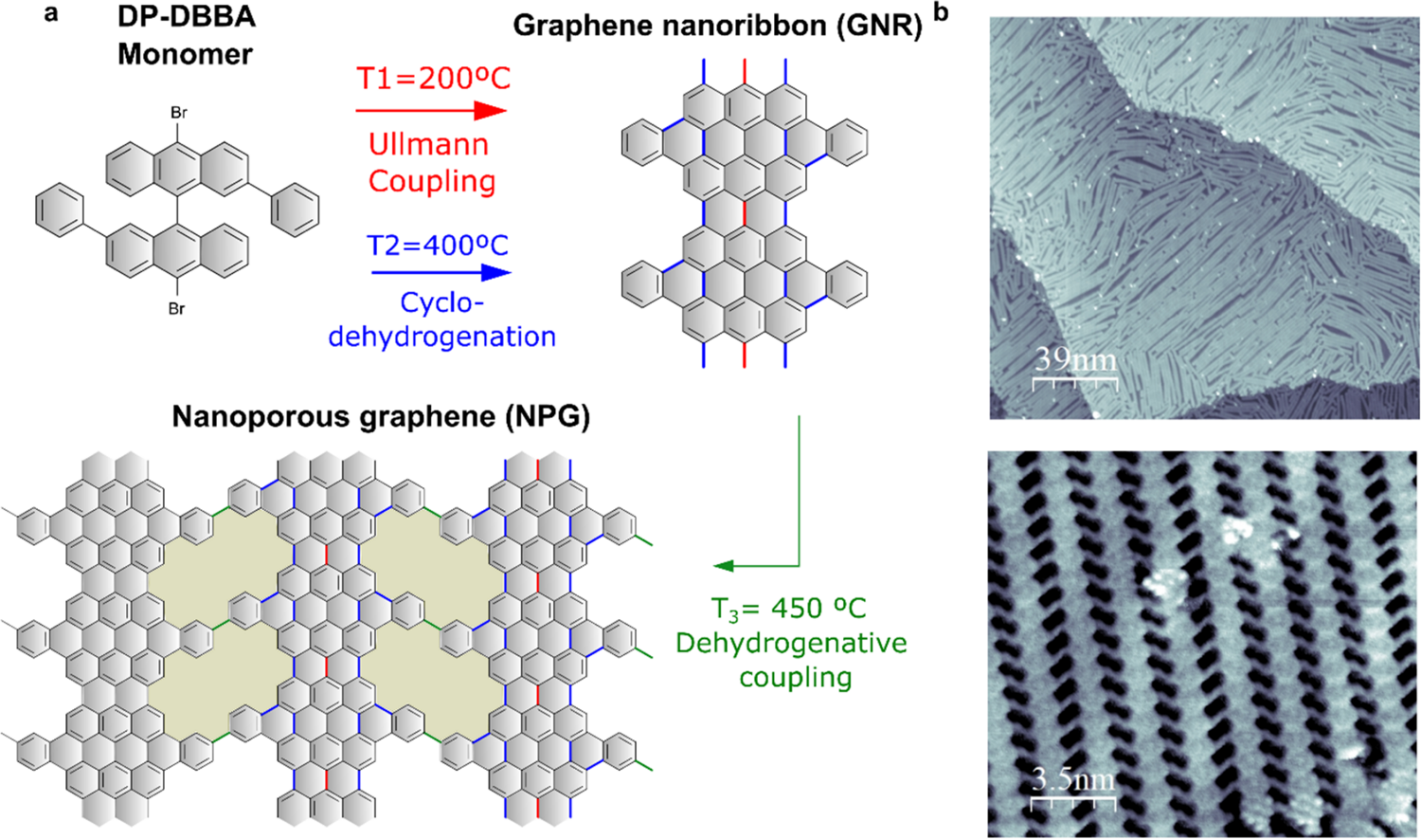
Bottom-up on-surface synthesis of nanoporous graphene. (a) Molecular structure of the DP-DBBA used as a precursor on this synthesis. 7–13-GNR was obtained after the Ullmann coupling and subsequent cyclodehydrogenation reactions induced at steps *T*_1_ and *T*_2_, respectively. At *T*_3_ the GNRs interconnect leading to the generation of the NPG structure. (b) Topographic STM images of the synthesized NPG. STM image parameters: overview (195 × 195 nm^2^, *I*_t_= 1.1 nA and *V*_s_= 2.0 V) and zoom-in (17.5 × 17.5 nm^2^, *I*_t_= 2.4 nA and *V*_s_= 0.8 V).

Pristine nanoporous graphene (NPG) was prepared following a previously reported bottom-up synthesis route.^1^ The process starts with a surface-assisted Ullmann coupling (*T*_1_ = 200 °C) of 10,10′-dibromo-2,2′-diphenyl-9,9′-bianthracene (DP-DBBA) to form long anthracene-based polymers, followed by the cyclodehydrogenative aromatization (*T*_2_ = 400 °C) of the intermediate polymeric chains to obtain a series of consecutive pairs of 7 and 13 C atom wide chains, and hence we label the ribbon 7–13-GNR (Figure 1a). Finally, the 7–13-GNR interconnect laterally via dehydrogenative coupling (*T*_3_ = 450 °C) to form NPG in a yield close to 100%.

The key ingredient for achieving a high-yield and long-range order observed in the final NPG product occurs as a consequence of the extraordinary length of the polymeric intermediates achieved and their parallel alignment driven by the reconstruction of the Au(111) herringbone substrate.^1,33,34^ The structure was characterized in each step of the hierarchical synthetic route by scanning tunneling microscopy (STM). The resulting NPG network exhibits domains as large as 70 × 50 nm^2^, containing atomically precise pores of 0.9 × 0.4 nm^2^, with an apparent height of 0.18 nm (Figure 1b).

**Figure 2:**
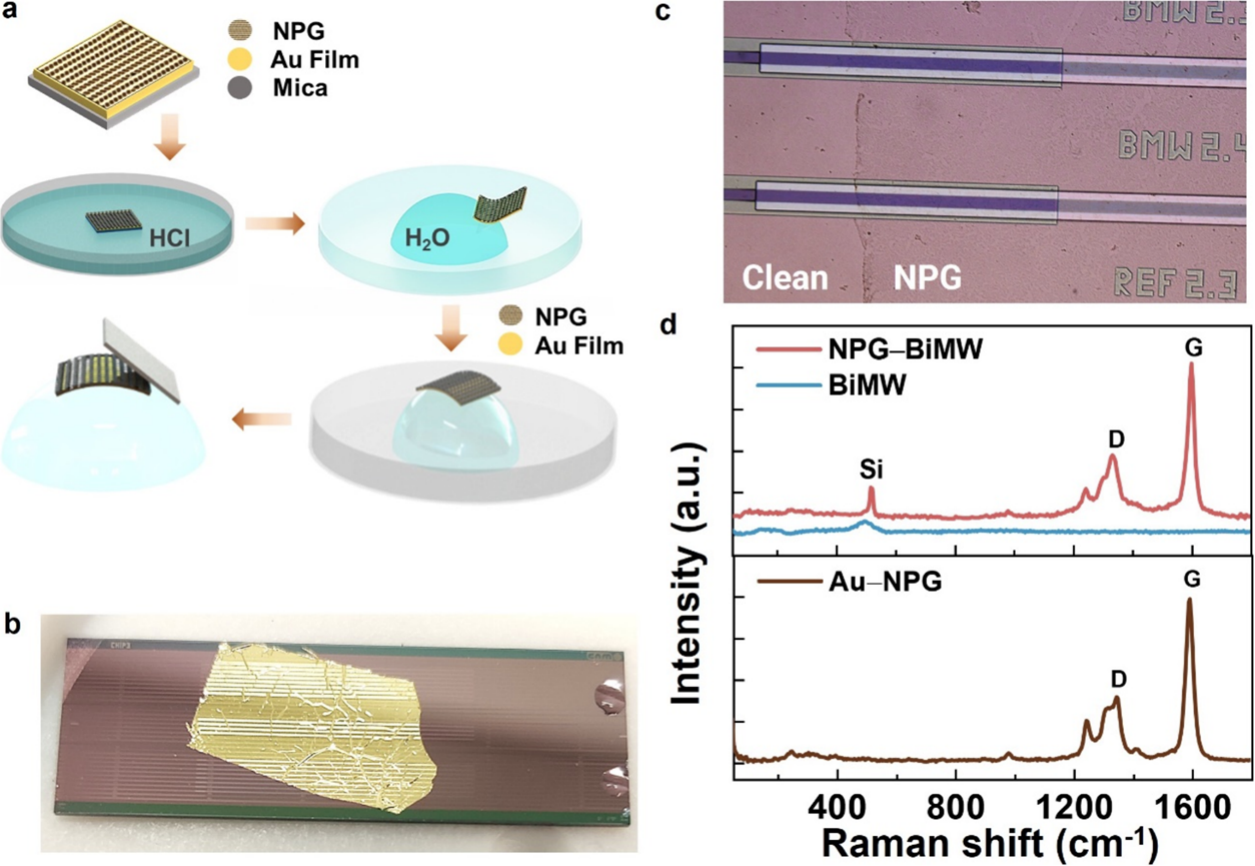
(a) Schematic of the polymer-free wet-transfer method used to integrate NPG onto the BiMW sensors. After separating the mica from the gold film-NPG, the latter is picked by the BiMW chip. (b) Photograph of the BiMW chip covered with NPG and a thin gold film over the sensing area. (c) Optical microscopy image of an NPG-coated waveguide after the thin gold layer has been removed. The NPG is visible due to a change in contrast. (d) Characteristic Raman spectra of bare BiMW (blue), NPG-BiMW (red), and Au(111)-NPG growth substrate (brown). The G- and D-bands are observed at 1601.0 and 1332.4 cm^–1^, respectively.

The integration of the NPG in the BiMW sensors is performed through a wet-transfer and polymer-free approach. In this method, the mica substrate of the NPG sample is detached in an HCl bath, leaving the thin gold layer-NPG floating in H_2_O. This gold layer, with the NPG on top, is then transferred to the sensor device through direct mechanical contact. Subsequently, the gold is etched away using a gold etchant solution, and the biosensor surface is thoroughly cleaned with several H_2_O rinsing steps followed by extended sonication. The schematics of the transfer process are shown in Figure 2a, resulting in a single layer of NPG uniformly covering all the waveguide devices (Figure 2b). After the gold etching procedure, the NPG-BiMW surface is characterized by optical microscopy and Raman spectroscopy, as depicted in Figure 2c,d. The transfer integrity of nanoporous graphene onto the waveguides was examined using optical microscopy, with high-contrast regions confirming the NPG presence (Figure 2c). The uncoated areas were quantified, showing that between 97–98% of the waveguide area is completely covered with NPG (Section B, SI). We characterized the NPG with Raman spectroscopy before the transfer, onto the gold film, and later onto the BiMW to confirm the result of the transfer process. Characteristic Raman features of NPG display G- and D-bands at 1601.0 and 1332.4 cm^–1^, respectively. The NPG Raman features are evident on the Au(111) growth substrate and after the transfer onto the BiMW (Figure 2d).

### Engineering and Optimization of the NPG-BiMW Photonic Biosensor System

**Figure 3:**
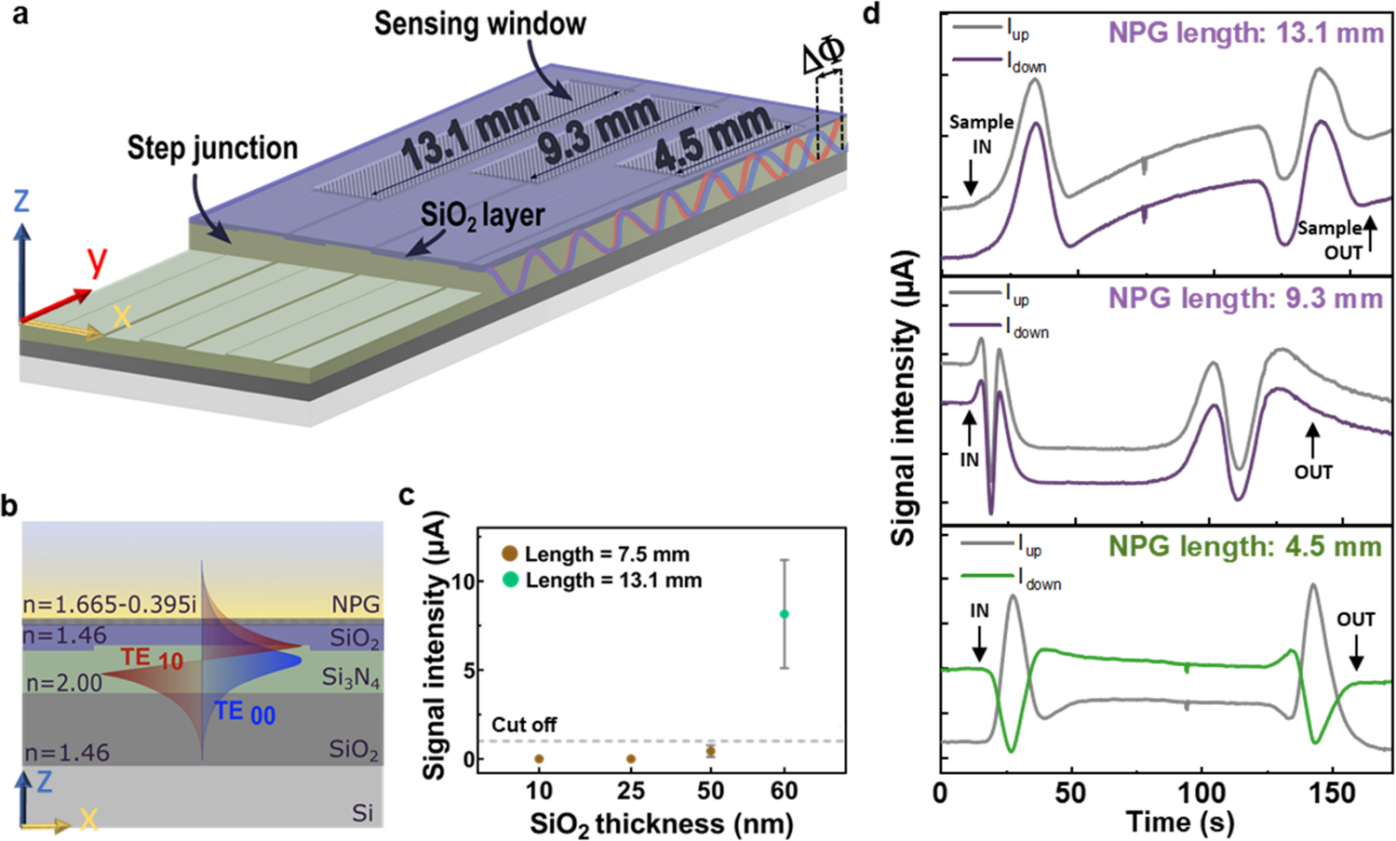
(a) Schematic of the BiMW device showing waveguides with varied lengths of the sensing area window (4.5, 9.3, and 13.1 mm). (b) Cross-section schematics of the NPG-BiMW device with the SiO_2_ spacer layer between the Si_3_N_4_ waveguide and the NPG coating. The refractive index of each material is indicated. The schematic is not at scale. (c) Output signal intensity measured for NPG-BiMW devices fabricated with variable thickness of the SiO_2_ spacer layer and lengths of the sensing area. Data points correspond to the mean and standard deviation of five different waveguides. (d) Temporal evolution of the interference pattern induced by a bulk RI change on three waveguides of the NPG-BiMW chip with different sensing lengths: 13.1, 9.3, and 4.5 mm. Arrows indicate the sample entrance to and exit from the sensing area.

Previous studies of silicon-based waveguides coated with graphene operating in the near-infrared region have shown high optical losses due to the evanescent field interacting with the graphene layer^35,36^ as it has a high imaginary part of the refractive index value (−1.52i).^37^ In contrast, the NPG displays a significantly lower imaginary part of the refractive index value (−0.395i),^38^ substantially reducing the light attenuation effect (Figure S4c, Section C, SI). Still, this value is far from negligible, and we could also expect limitations in light propagation on the NPG-BiMW system.^39^ To understand and minimize this effect, we performed numerical mode analysis and evaluated the attenuation constant (α) of the NPG-BiMW, using the theoretical refractive index (*n* = 1.665–0.395i) of the NPG layer^38^ (Figure S4c, Section C, SI). The study was performed for the fundamental mode (TE_00_) in a cross-section waveguide, resulting in an attenuation constant α = 0.85 dB/cm for bare BiMW and α = 80.6 dB/cm for the NPG-BiMW, indicating an increase in the optical losses of the waveguide induced by the presence of the NPG. To reduce this attenuation, we proposed the strategy of including a spacer layer of SiO_2_ (*n* = 1.46) between the NPG and the Si_3_N_4_ waveguide, which would act as a buffer due to the negligible imaginary part of its refractive index (Figure 3b).^40^ We studied the variation of α with the increase in the SiO_2_ thickness (ranging from 10 to 100 nm) and the results show a total attenuation reduction of 62% (21.2 dB/cm) for the NPG-BiMW with 60 nm thick SiO_2_ layer, respect to the NPG-BiMW device without SiO_2_ layer. However, it should be noted that increasing the spacing layer thickness may reduce the overall sensor sensitivity due to a decrease in the evanescent field intensity in contact with the sample. Additionally, previous works have also suggested that the effective coverage length of graphene onto photonic Si_3_N_4_ waveguides also influences light propagation, indicating that the accumulated overlap of the evanescent field over a certain distance might result in complete light attenuation.^36^ This could be especially relevant for the design of our bimodal interferometer, given that the two propagating modes (fundamental and first-order modes) have different evanescent field penetrations, and the different interactions with the NPG layer might affect the sensing interferometric behavior.

Based on the numerical calculations, BiMW devices were modified by adding a SiO_2_ spacer waveguide coating of different thicknesses (10, 25, 50, 60, and 100 nm) and also by reducing the sensing window lengths (4.5, 7.5, 9.3, and 13.1 mm) (Figure 3a–c). The reduction of the sensing window was performed by partially passivating the sensing area with a thick SiO_2_ cladding (360 nm), which fully covers the evanescent field penetration and avoids contact with the sample (Figure S7, Section D, SI). First, we compared the phase shift (ΔΦ) values of the different SiO_2_-coated BiMW to in-solution RI variations (Δ*n*). From this refractometric sensing evaluation, we determined that the maximum SiO_2_ thickness suitable for effective refractometric sensing is around 50–60 nm, providing a competitive sensor resolution in the range of 10^–6^ RIU (Figure S8b, Section D, SI). Next, we evaluated the signal intensity of the light propagated along the NPG-BiMW with different SiO_2_ thicknesses (10, 25, 50, and 60 nm) and sensing window lengths (7.5 and 13.1 mm) (Figure 3c). A cutoff value of 1 μA was established as the minimum signal intensity to ensure sufficient signal-to-noise ratio (SNR) for biosensor evaluation. With these conditions, we observed that even with a relatively long sensing window (13.1 mm), the 60 nm spacer layer was sufficient to allow light propagation with an appropriate output intensity. Furthermore, because of the structural modifications made to allow light propagation with the new NPG-BiMW sensor, additional adjustment of the biosensor was performed to adapt the optical setup to the optimum working wavelength. Based on calculations of the effective refractive index difference (Δ*n*_eff_) between the fundamental and the first-order modes, the ideal wavelengths for our new biosensor would fall between 532–632 nm, therefore, we selected a 532 nm laser diode as a light source (Figure S5a, section C, SI).

Finally, the interferometric behavior of the NPG-BiMW system was assessed. Considering the differential attenuation effect of the NPG over the evanescent field of the two light propagating modes, we experimentally optimized the sensing window length of the device to ensure an interferometric output. Figure 3d shows the sensor responses obtained from several waveguides of the same chip but with different sensing window lengths (13.1, 9.3, and 4.5 mm, respectively) upon the introduction of a different refractive index solution (0.8% DMSO, Δ*n* = 0.01062 RIU). As can be observed, only the waveguide with a 4.5 mm sensing length allowed the formation of the expected interferometric pattern at the output (i.e., *I*_up_= −*I*_down_). On one hand, these results confirm that the accumulated contact of the evanescent field with the NPG induces different attenuation effects on the two modes of light, which directly affects their propagation condition and subsequently the interferometric behavior of the device. By selecting the 4.5 mm sensing length, we complete the optimized engineering of the new NPG-BiMW system, demonstrating a fully operative interferometric sensor coated with a presynthesized NPG template for direct cross-linking of bioreceptors. To further confirm the reliability of our results, we characterized five different sensor devices with lengths of 4.5 mm, as well as several with shorter lengths. All devices tested exhibited interferometric signals at the output.

### Demonstration of the NPG-BiMW Biosensor for Label-Free Biomolecular Analysis

**Figure 4:**
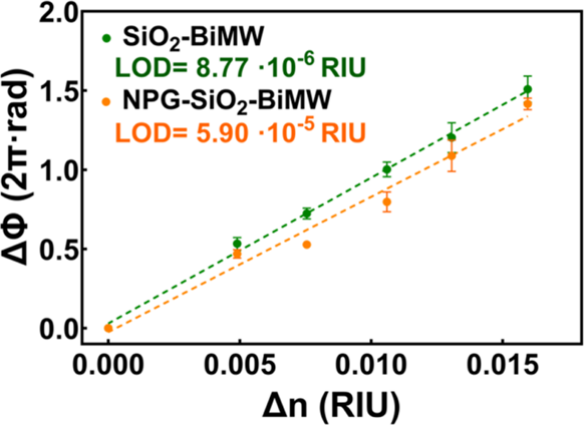
Bulk sensitivity calibration curve of the SiO_2_-coated BiMW sensor (green) and the NPG-BiMW sensor (orange). The data points correspond to the phase shift (ΔΦ) value obtained with triplicate measurements of increasing DMSO dilutions (0.4–1.2% DMSO).

Before addressing the NPG biofunctionalization, the NPG-BiMW was evaluated as a refractometric sensor by performing bulk sensitivity calibration to changes in the refractive index (Δ*n*) of the medium. The bulk calibration was performed for the SiO_2_-coated BiMW device with and without the NPG integration. Figure 4 depicts the phase shift (ΔΦ) measured inflow for each concentration of DMSO (ranging from 0.4–1.2%, Δ*n* = 0.00495–0.01637 RIU). The device sensitivity, determined by the slope of the curve, resulted in 92 2π·rad/RIU for the BiMW device without NPG and 84.5 2π·rad/RIU for the NPG-BiMW. These values are in good agreement with our preliminary simulations (Figure S6, Section C, SI), indicating a slight but negligible detrimental effect of the NPG on the BiMW sensor sensitivity. Likewise, the limit of detection (LOD), corresponding to the smallest detectable signal and calculated as the signal corresponding to three times the background noise (i.e., standard deviation of the baseline), was determined at 8.77 × 10^–6^ RIU for the SiO_2_ coated BiMW and 5.90 × 10^–5^ RIU for the NPG-BiMW. This increase in the LOD for the NPG-BiMW is mainly attributed to the higher background noise (standard deviation of the baseline) observed for the NPG-BiMW (σ = 1.7 × 10^–3^) compared to the SiO_2_ coated BiMW (σ = 0.26 × 10^–3^). The increased background noise of the NPG-BiMW sensor could be attributed to the increase in the attenuation losses of the waveguide after NPG coating, as the SNR is generally proportional to the output signal intensity.^41^ Notwithstanding, our NPG-BiMW sensor shows a remarkable refractometric sensitivity within the same order of magnitude as other silicon nanophotonic sensors, like Mach–Zehnder interferometer or ring resonators (ranging between 10^–5^ and 10^–7^ RIU),^42,43^ and it might be superior to other label-free optical sensor technologies, like plasmonics or photonic crystals, generally ranging between 10^–4^ and 10^–6^ RIU.^44−46^

**Figure 5:**
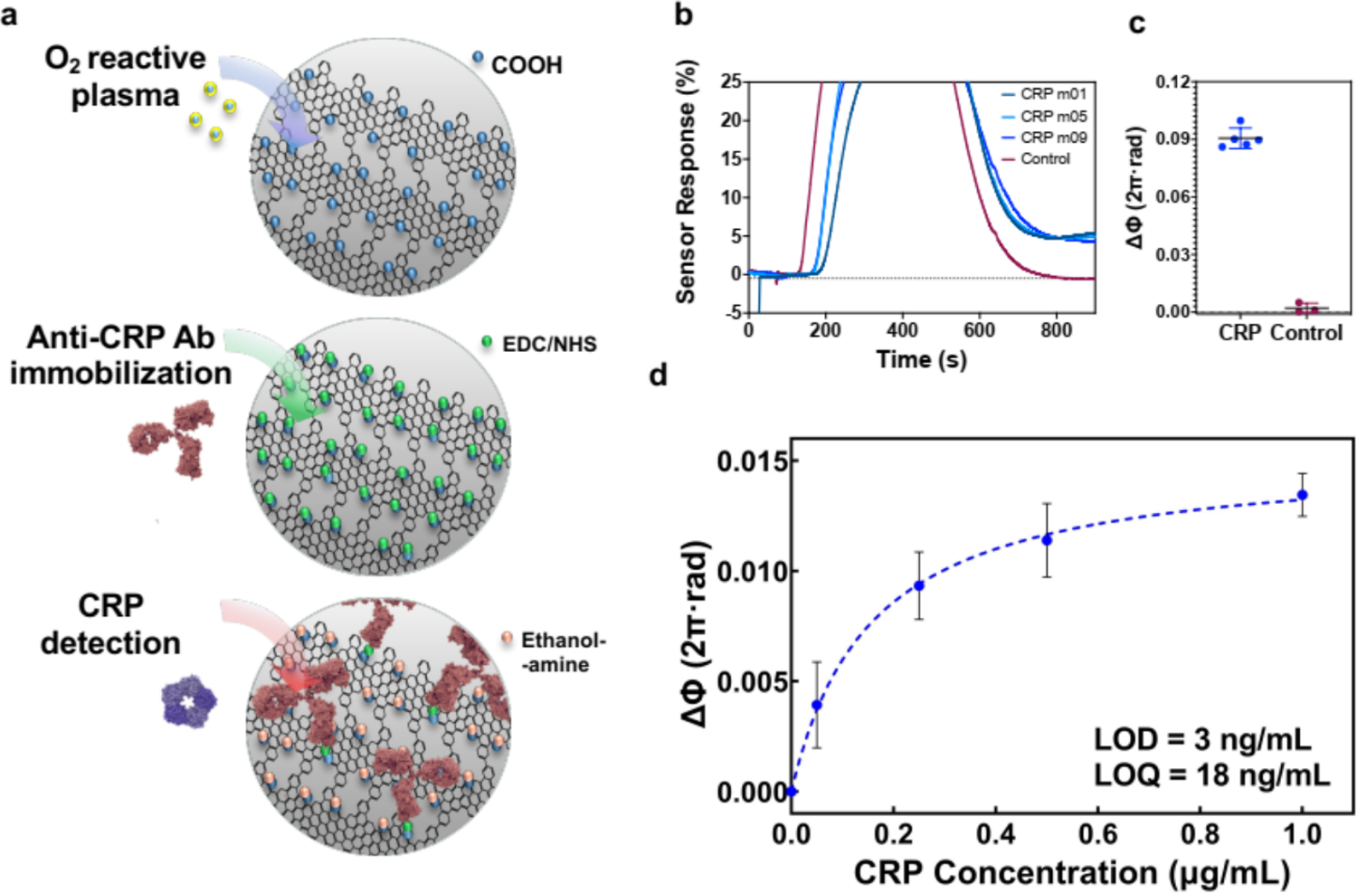
(a) Schematics of the NPG biofunctionalized structure and the subsequent covalent immobilization of antibodies through EDC/NHS cross-linking to COOH groups introduced at the NPG structure. The surface is blocked with ethanolamine to avoid unspecific bindings. (b) Overlaid real-time sensorgrams for the detection of CRP at the same concentration and of the negative control (BSA) for specificity test. (c) Statistical comparison between specific and unspecific samples measured in the same NPG-BiMW sensor device. (d) Calibration curve for CRP detection at different concentrations (0.05–1 μg/mL). Each data point corresponds to the mean ± SD of the duplicate measurements performed on different NPG-BiMW sensor devices.

Lastly, we carried out the functionalization of the NPG-BiMW biosensor and demonstrated its performance for relevant biomolecular analysis. For that, we addressed the immobilization of monoclonal antibodies on the NPG scaffold for the specific detection of C-reactive protein (CRP). CRP is an acute-phase biomarker protein whose levels in the blood increase dramatically in response to inflammation, infection, and tissue damage. It is commonly targeted for diagnosing infections and sepsis, chronic inflammatory diseases, assessing cardiovascular risk, monitoring surgical complications, and evaluating therapeutic responses.^47,48^ The procedure is illustrated in Figure 5a. Taking advantage of the high reactivity of the nanopore regions, we introduced oxide-based reactive chemical groups (COOH, COC, CHO, OH) within the NPG scaffold by exposing the NPG-BiMW chip to a few seconds of O_2_ plasma process (12 s). The method was optimized to minimize defects at the NPG structure, by characterizing and confirming the structural integrity of the graphene through XPS, and the appearance of oxygen-containing functional groups was also verified (Figure S10, section E, SI). The subsequent anchoring of antibodies was done through the well-established carbodiimide-based chemistry, which enables the cross-linking of COOH groups but also COC and CHO to abundant primary amines (NH_2_) in the antibody. This biofunctionalization process (Figure 5a) begins by injecting a 0.2 M EDC/0.05 M NHS solution in MES buffer. This step replaces the NPG oxygen-functional groups with highly reactive NHS esters. To preserve the stability of the NHS esters for subsequent antibody binding, Milli-Q water is used as running buffer, providing a slightly acidic pH that is more suitable than conventional neutral-pH buffers (e.g., PBS). Immediately after EDC/NHS activation, an anti-CRP antibody solution (20 μg/mL in PBS, pH 7.4) is flowed over the sensor, enabling covalent binding to the active NHS groups and forming stable amide bonds. Finally, unreacted NHS groups are inactivated with an ethanolamine solution. The anti-CRP antibody immobilization process was monitored in real-time (Figure S11a, Section F, SI), showing a significant phase shift for antibody binding to the NHS-NPG surface. The successful formation of a covalent bond and the biolayer stability were proven by injecting a low-pH solution (HCl 10 mM, pH 2), which would disrupt any noncovalent interactions, showing a decrease in the sensor signal (data not shown). Upon NPG biofunctionalization was completed, the running buffer was changed to PBS pH 7.4 to ensure the biological activity of antibodies and maximum detection efficiency. Sequential detection cycles of CRP were performed to further assess the robustness of the antibody-functionalized NPG-BiMW sensor surface. CRP samples diluted in PBS buffer at a fixed concentration (1 μg/mL) were flowed over the biosensor, followed by a 1 min regeneration step using a 10 mM HCl solution. This short regeneration treatment with a low-concentration acidic solution effectively disrupts the antigen–antibody interaction while preserving the antibodies’ biological activity, allowing for subsequent detection measurements.^49,50^ The experiment was repeated up to 9 cycles, showing a similar biosensor response for all cases (Figure 5b,5c). This confirmed the stability of the biofunctionalized NPG scaffold and the good reproducibility of the biosensor assay. Besides, the detection specificity was evaluated by introducing a nontarget protein of a similar molecular weight (BSA) as negative control, which did not produce any significant biosensor response (i.e., baseline returns to initial position) (Figure 5b,c).

Finally, the analytical sensitivity of the NPG-BiMW biosensor was evaluated by carrying out a CRP detection calibration. Different samples of CRP at increasing concentrations (0.05–1 μg/mL) were evaluated by duplicate and the biosensor response was plotted and fitted to a one-site binding saturation model (Figure 5d). Additionally, sensor quality controls were performed intermittently by testing a CRP sample of 1 μg/mL. The LOD was determined at 3 ng/mL, which is in the same order of magnitude as those obtained with conventional BiMW sensors biofunctionalized through silanization procedures (7 ng/mL),^51^ and falls within the typical range of LODs achieved with label-free photonic biosensors for direct, nonamplified CRP detection (0.1–10 ng/mL).^52^ It is worth noting that the LOD achieved by the NPG-BiMW is well below the clinical cutoff levels required for the diagnostic detection of CRP in patient samples,^53^ therefore showing promising technology application prospects within the medical field.

## Conclusions

In this work, we have combined bottom-up synthesized nanoporous graphene with a silicon-based nanophotonic biosensor, representing a major advancement in simplifying biofunctionalization strategies for biosensor technology. This integration harnesses the high sensitivity of bimodal waveguide interferometers and the large functional surface area of nanoporous graphene to create highly sensitive, selective, and robust biosensors for the direct immunoassay detection of CRP, a crucial inflammatory biomarker in the clinical diagnosis of infections and sepsis. Our biosensor proved to be highly specific and sensitive, with an excellent limit of detection (LOD = 3 ng/mL) comparable to similar devices, below the sepsis diagnosis cutoff levels. The NPG biointerface remained stable and reproducible, exhibiting low variation in the detection, even after 9 regeneration cycles.

With this innovation proof-of-concept and experimental validation, our work opens new avenues in designing and integrating NPG as atomically precise biofunctionalization scaffolds. Controlling pore size and geometry during on-surface synthesis can benefit the bioreceptor density and distribution control, with prospective applications for the optimal detection of different biological targets, such as small molecules, proteins, nucleic acids, or pathogens and cells. Furthermore, the possibility to intrinsically functionalize the NPG pore regions with specific reactive groups, such as amines or thiols, may greatly expand the portfolio of cross-linking strategies that can be easily adapted to a wide variety of bioreceptors, including antibodies, peptides, DNA probes, or aptamers.

## Supporting Information Available

Experimental details for the determination of the sensing area length, and the length of the NPG transfer; additional analysis on the numerical calculations performed for the optimization of the NPG-BiMW device; description of the strategy for SiO_2_ layer coating and calculations of the device sensitivity; XPS characterization data for the oxygen plasma treatment on NPG; and additional description of the biosensor assay conditions applied on the NPG (PDF)
