# Supplementary material for: Nanoporous Graphene Integrated onto Bimodal Waveguide Biosensors for Detection of C-Reactive Protein
Source: ACS Appl Nano Mater. 2025 Jan 10;8(3):1640–8. doi: 10.1021/acsanm.4c06716 (PMC11773638; doi:10.1021/acsanm.4c06716)
Supplement: Supplementary file 1 — an4c06716_si_001.pdf [file an4c06716_si_001.pdf]

Supporting Information

Nanoporous Graphene Integrated onto Bimodal Waveguide Biosensors for Detection of C-Reactive Protein

Bárbara Lisboa[1,2], Maria Soler[1], Rukmani Singh[1], Jesús Castro-Esteban[3], Diego Peña[3,4], Aitor Mugarza[2,5], Laura M. Lechuga[1], César Moreno[2,6]**

1 Nanobiosensors and Bioanalytical Applications Group (NanoB2A), Catalan Institute of Nanoscience and Nanotechnology (ICN2), CSIC, BIST and CIBER-BBN, 08193 Bellaterra, Barcelona, Spain

2 Atomic Manipulation and Spectroscopy Group (AMS), Catalan Institute of Nanoscience and Nanotechnology (ICN2), CSIC and BIST, 08193 Bellaterra, Barcelona, Spain

3 Centro de Investigación en Química Biolóxica e Materiais Moleculares (CiQUS) and Departamento de Química Orgánica, Universidade de Santiago de Compostela, 15782 Santiago de Compostela, Spain

4 Oportunius, Galician Innovation Agency (GAIN), 15702 Santiago de Compostela, Spain

5 ICREA – Institució Catalana de Recerca i Estudis Avançats, 08010 Barcelona, Spain

6 Departamento de Ciencias de la Tierra y Fisica de la Materia Condensada, Universidad de Cantabria, 39005 Santander, Spain

Corresponding Author

E-mail: maria.soler@icn2.cat; cesar.moreno@unican.es

TABLE OF CONTENTS

A. Description of the BiMW sensor technology and sensing mechanism S-2

B. Determination of the length of the sensing area covered by NPG................................ S-4

C. Numerical calculations for the optimization of the NPG-BiMW device......................... S-4

D. Strategy for SiO_2 spacer layer coating & experimental sensitivity................................ S-8

E. X-ray photoemission spectroscopy (XPS) characterization.. S-10

F. Bioassay conditions on the NPG layer.. S-12

A. Description of the BiMW sensor technology and sensing mechanism

The BiMW sensor consists of a single straight waveguide with a sensing mechanism based on the interference between two guided modes of the same polarization (see Fig S1). Briefly, the BiMW sensor has a first waveguide area exhibiting single-mode behavior, where only the fundamental mode of the light is propagating. After some distance, the core thickness of the waveguide is increased (1-3 nm height rib), and the light modes are split in the fundamental and first order. Both modes keep traveling through until the output of the sensor chip. A sensing window (15 mm length) is opened at the bimodal part of the waveguide to allow the evanescent field to interact with the external medium. On the sensor surface, a bioreceptor layer is immobilized. When a biorecognition process occurs in the sensor area, the refractive index of the surface changes. This variation of the external refractive index affects the effective refractive index of the modes.

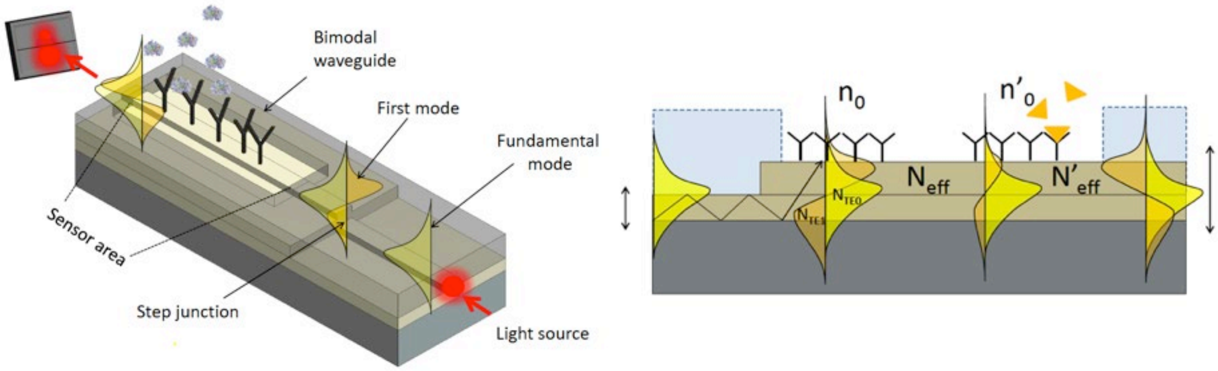

Figure S1. Schematic illustrations of the BiMW sensor technology and sensing principle.

Due to the different confinement of each mode in the core of the waveguide, they are differently affected by the change in the external refractive index, creating an interference pattern at the exit of the waveguide according to the equation:

$$\Delta\Phi = 2\pi \frac{L}{\lambda}\left(n_{eff_{00}} - n_{eff_{10}}\right)$$

where L is the sensor area's length, $\lambda$is the working wavelength, and *n* is the effective refractive index at the surface of the waveguide.

The BiMW sensor device implemented in this work uses a 532 nm diode laser polarized in the transverse electrical (TE) state as the light source. The beam propagates through a Faraday isolator lens (LP-532 Thorlabs, Newton, New Jersey, USA), a 40x microscope objective (Olympus PLN 40× Objective, Edmund Optics, Barrington, New Jersey, USA), and is confined on the rib waveguide with power of 7.9 mW. A two-section photodetector (I_{up} and I_{down}) (Hamamatsu Photonics, Hamamatsu, Japan) records the output

sensor signal. Light exiting the waveguide generates currents I_{up} and I_{down} in the upper and lower sections of the photodetector, respectively. The values of the currents are proportional to the phase variation ($\Delta\Phi$) of the signal and dependent on the signal visibility (V), according to the expression:

$$\frac{I_{up} - I_{down}}{I_{up} + I_{down}} \propto \mathrm{V}\cos(\Delta\Phi(t))$$

The photodetector signals are acquired through an acquisition card (National Instruments, US) and processed with an in-house developed Labview software (Fig. S2).

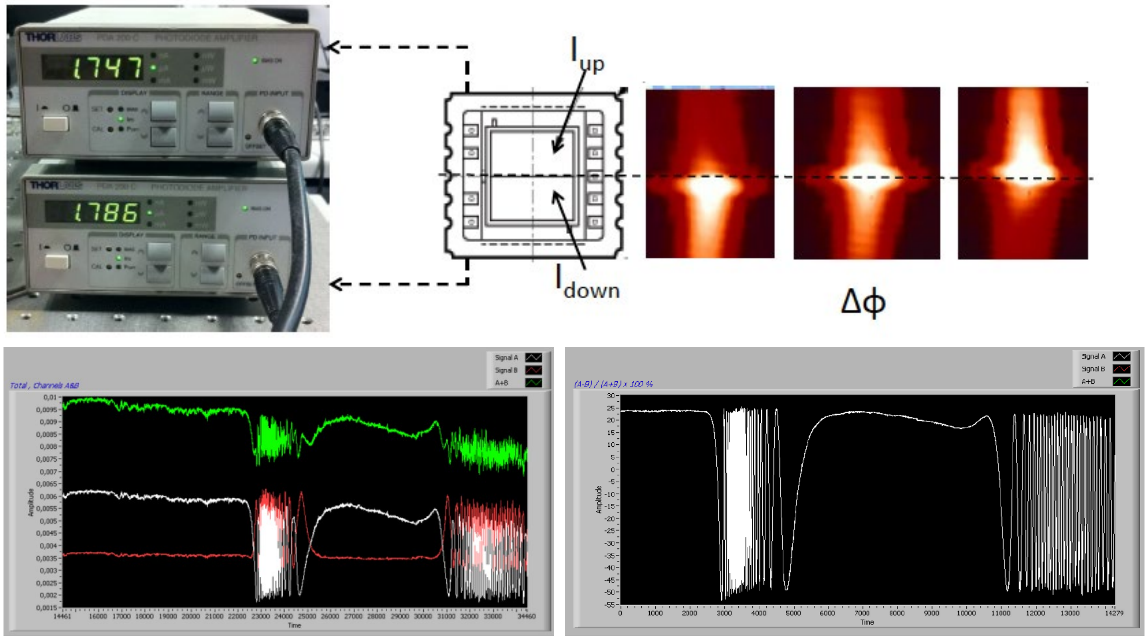

Figure S2. Schematics of the output signal processing of a BiMW device (up), and screenshots of the software for raw data visualization and processing.

The temperature of the BiMW device is stabilized by a Peltier thermoelectric cooler (Peltier element TEC3-2.5) (Thorlabs, New Jersey, US) connected to a temperature controller with a precision of 0.01 °C. A fluidic system composed of a five-channel polydimethylsiloxane (PDMS) microfluidic platform (channel dimensions= 1.25 mm width x 500 µm height), a peristaltic pump (Miniplus, Gilson, US), and a 6-port injection valve (VICI, Texas, US), is mounted hermetically on top of the BiMW to allow a continuous flow rate of the running buffer and the injections of the samples to be analyzed.

B. Determination of the length of the sensing area covered by NPG.

The length of the transferred NPG in the sensing area was determined by optical microscopy imaging from the contrast produced by NPG. Figures S3a and b display the limits comprising the NPG area, where the

green lines represent the sensing area limits and the pink lines the NPG transfer limits. Figure S3c was used to calculate the number of defects after the NPG transfer.

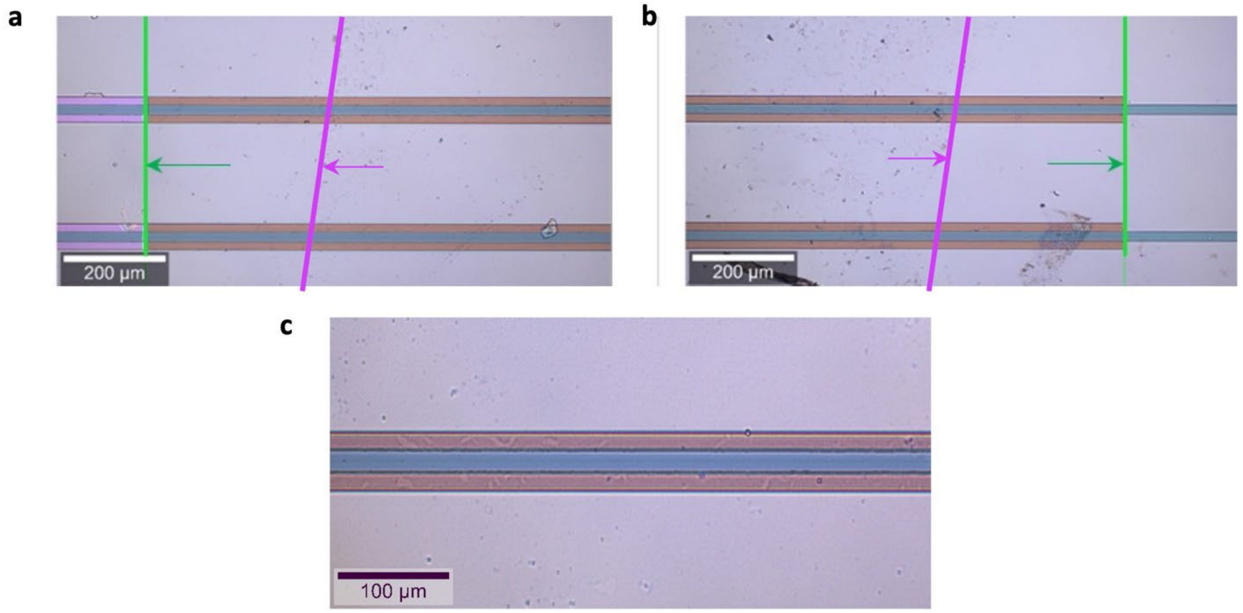

Figure S3. Characterization of the sensing window length using calibrated optical microscopic images. a, b) The green lines define the start and end points of the sensing area, and the pink lines the NPG transfer limits. c) Image captured in the middle of the waveguide covered with NPG.

C. Numerical calculations for the optimization of the NPG-BiMW device

To investigate the optical characteristics of the NPG-BiMW device, we performed numerical calculations based on modal analysis employing a finite element method-based simulation tool: wave optics module of COMSOL Multiphysics. The computational two-dimensional cross-section model of the NPG-BiMW structure is described in Figure S4a. The supported mode profiles by the waveguide, which are fundamental and first-order modes of the transverse electric field (i.e., TE_{00} and TE_{10}) are explored for further calculations as shown in Fig. S5a. The effective refractive index, one of the most basic optical parameters for estimating optical characteristics, is exposed for both modes considering the NPG layer and without the NPG layer on the conventional BiMW structure, as shown in Fig. S4a. The normalized electric field distribution through the propagating modes for the NPG-BiMW device is represented in Fig. S4b, which indicates proper overlapping of both modes and helps to understand that the SiO_2 layer reduces the effective penetration depth of evanescent wave (EW) in the cladding by its thickness.

To evaluate the optical losses of the waveguide device, we characterized the attenuation constant (α_0) using the following equation:[1]

$$\alpha_0 = 8.686 \times \frac{2\pi}{\lambda} \times Im\,(N_{eff}) \times 10^4 \text{ (dB/cm)} \quad (1)$$

where α_0 is calculated from the imaginary value of the effective refractive index (Im (N_{eff})) of the TE_{00} mode at a wavelength of 660 nm. Eq. (1) indicates that a higher value of Im (N_{eff}) results in larger attenuation of light in the waveguide. The value of Im (N_{eff}) is observed higher for the NPG-BiMW compared to the pristine BiMW structure, indicating increased optical losses to occur at the NPG-BiMW interface. The inset values in Fig. S4c represent the BiWM at different stages of the NPG integration: i) for the bare BiMW is 0.85 dB/cm, ii) the NPG-BiMW without added spacer layer is 80.6 dB/cm and, iii) the final device NPG- SiO_2-BiMW with 60 nm thickness of SiO_2 is 21.2 dB/cm. The advantage of using nanoporous graphene over graphene, in terms of light attenuation, could clearly be observed from simulations displayed in Figure S4c. Graphene-BiMW displays a total attenuation constant of ca. 500 dB/cm while NPG is ca. 80 dB/cm, i.e. 6 times larger than NPG. It is worth noting that factor 6 remains unaltered with the SiO_2 thickness while the SiO_2 thin film itself doesn't change significantly the attenuation constant of the BiMW.

Considering the larger value of α_0 due to the integration of the NPG layer on BiMW, we estimated the light propagation distance in the waveguide before it gets fully absorbed and denoted it by effective propagation distance (L_{eff}). The L_{eff} was calculated by using Eq. (2)[2]. Fig. S8d, displays calculated L_{eff} as a function of NPG layer length (L_{NPG}) on NPG-BiMW with the SiO_2 thickness varying between 0 to 100 nm.

$$L_{eff} = \frac{1 - \exp^{-\alpha_0 L_{NPG}}}{\alpha_0} \quad (3)$$

α_0 is the attenuation constant extracted from Fig. S4c for NPG-BiMW device, and the L_{NPG} varies from 0 to 4 mm. From the plot (Fig. S8d), the length of the NPG needed to reach the saturation point of the plot is 0.25 mm for 0 nm of SiO_2, 2 mm for 50nm of SiO_2, and more than 4mm for 100 nm of SiO_2. The saturation point increases 8 times for the NPG-BiMW waveguide with 50 nm of SiO_2 compared with the device without the SiO_2 layer. This result is similar to what was obtained experimentally, as for an NPG-BiMW without SiO_2, the light is fully absorbed when covered with 1.5 mm of NPG length (data not shown), and for the NPG-BiMW waveguide with 50 nm of SiO_2, we enter the saturation region with 7.5 mm of NPG length, the increase is in the same order of magnitude as the simulated results (i.e. an increase of 5 times).

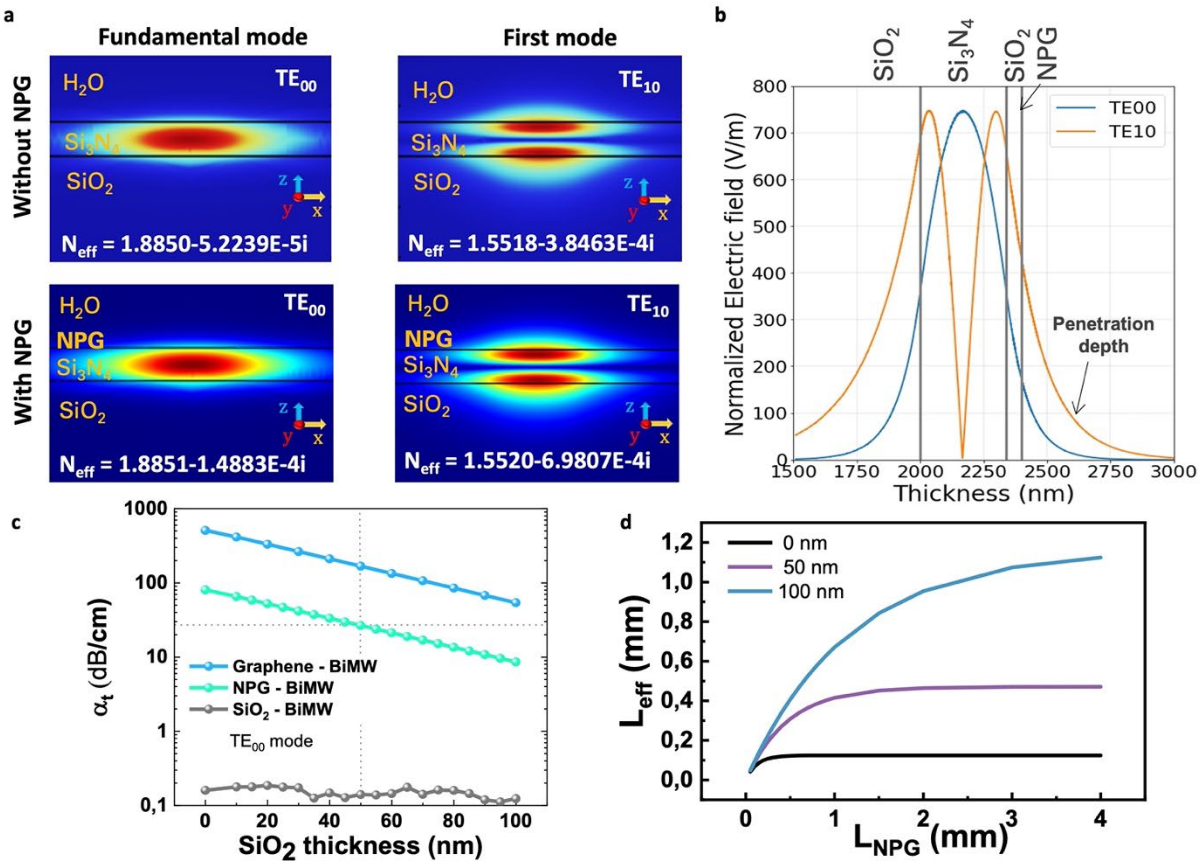

Figure S4. a) Mode profiles of the fundamental (TE_{00}) and first order (TE_{10}) transverse electric field polarization at the cross-section of the sensing region of the device with and without the NPG layer. b) Normalized electric field intensity distribution of each mode on the NPG-BiMW cross-section. c) Total attenuation (α_t) for SiO_2-BiMW, NPG- SiO_2-BiMW, and graphene- SiO_2-BiMW as a function of SiO_2 thickness (ranging from 10 to 100 nm). d) Effective propagation length of light as a function of the NPG length on the NPG-BiMW without SiO_2 and with SiO_2 (50 and 100 nm) waveguide.

To respond to the critical effects of hetero-modal interferometers, the working wavelength of the NPG-BiMW was evaluated in terms of the effective refractive index difference in the function of wavelength. Thus, a critical point arises when the following condition is verified:

$$\frac{\partial}{\partial \lambda}\frac{\Delta n_{eff}(\lambda)}{\lambda} = 0$$

where the Δn_{eff} represents the difference between the real part of the effective refractive index of the TE_{10} and TE_{00} modes. The critical effects of interferometric sensors are described more in detail by Dante et al.[3] The definition implies that close to the minimum or maximum of the function, the sensitivity of the interferometric sensor deviates from potential ultra-high sensitivity detections, limited only by the system noise, and optimal interferometric behavior.[4] The result of the definition described is represented in Fig.

S5a for the bare BiMW and the NPG-BiMW. The simulations derive from the obtained effective refractive difference for the cross-section described above, with water at the sensing medium. The result of the critical wavelength for a bare BiMW is 685 nm, which is in good agreement with the value published.[3] Whereas, for the optimized NPG-BiMW device, the critical point shifts to 575 nm. However, due to the limited commercial diode laser available in the region between 540 - 620 nm, we decided to optimize the working wavelength to a diode laser of 532 nm wavelength.

After integration of NPG on the BiMW with the 532 nm wavelength, a typical destructive interference pattern is observed (Fig. S5b) when inducing an Δn change in the sensor surface, while with other available lasers in the lab (660 nm wavelength) the device did not respond correctly (Fig. S5c). In this regard, experimentally validated the numerical calculations showing the device's dependence on the light beam wavelength to achieve an interferometric response (Fig. S5b-c).

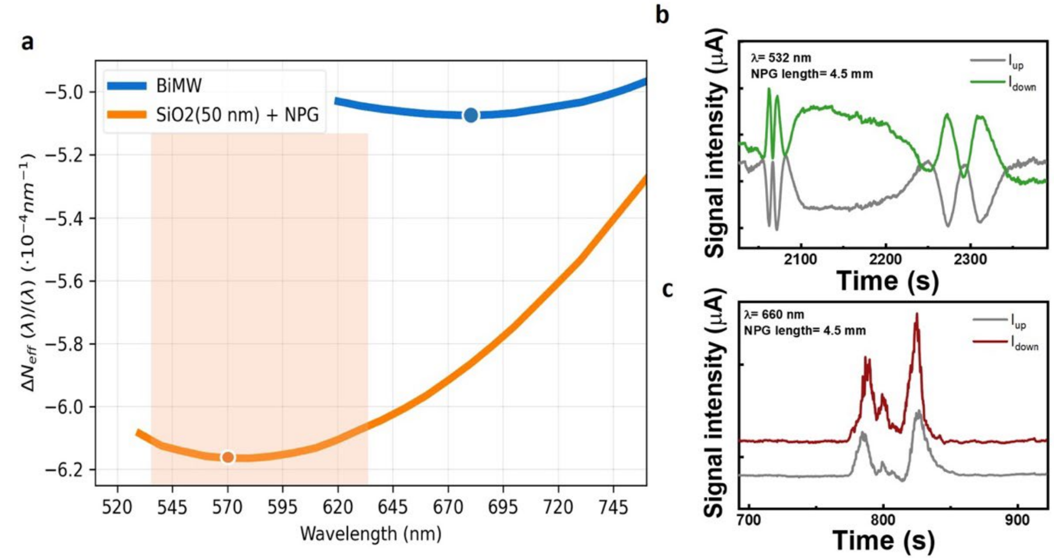

Figure S5. a) Study of the effective refractive index variation in function of the wavelength for bare BiMW device and optimized NPG-BiMW device to find the critical wavelength of operation. The plots of the temporal evolution of the interference pattern obtained for the same NPG-BiMW device tested with laser wavelengths of b) 532 nm and c) 660 nm, showing, respectively, destructive and constructive interferometric behavior.

The bulk sensitivity (S_{bulk}) is theoretically calculated using the following expression:

$$S_{bulk} = \frac{\partial(\Delta n_{eff})}{\partial n}$$

where the Δn_{eff} represents the difference between the real values of the effective refractive index of the TE_{10} and TE_{00} modes. The variation on the real effective index is related to the phase shift observed during RI variation. A parametric swept function was added to define the sample medium refractive index variation (∂n) from n= 1.333 to 1.393 in a sweep of 0.001 and observed the corresponding Δn_{eff} for each propagation mode. The bulk sensitivity value was calculated for different SiO_2 thicknesses of 25, 35, 50, and 100 nm. As expected, in Fig. S6 the sensitivity is decreasing with the SiO_2 thickness due to the reduced interaction of the sample with the evanescent wave field.

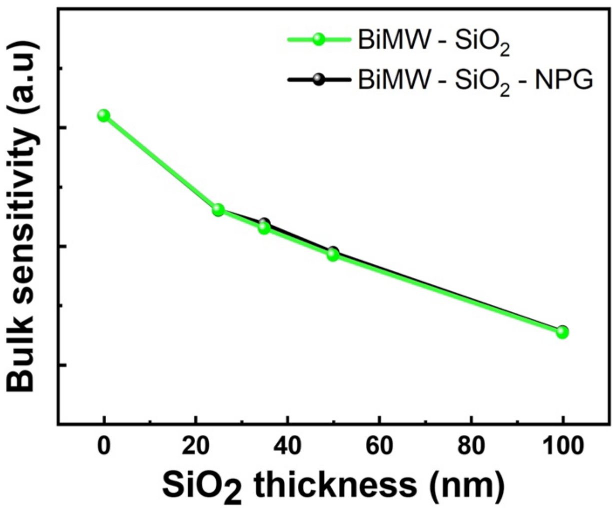

Figure S6. Theoretical bulk sensitivity of the BiMW for different SiO_2 thicknesses devices with and without NPG.

D. Strategy for SiO_2 spacer layer coating & experimental sensitivity

The BiMW is modified for the integration of NPG in the sensor area, a SiO_2 layer is evaporated onto the Si_3N_4 core before the NPG transfer, as represented in Fig. S7a-b. The SiO_2 layer is employed either to reduce the sensing area length or to define the spacer layer. Firstly, the length is reduced by blocking the evanescent field with a 300 nm thickness SiO_2 layer. The sensing area length is reduced according to the range of lengths tested (L= 4.5 – 13.1 mm) by employing a mask during evaporation. The length of the optimized device is 4.5 mm. To complete the strategy, in the final sensing area region, a spacer layer of thickness ranging from 10 to 100 nm is evaporated above the Si_3N_4 core to reduce optical attenuation effects, the thickness of this layer was optimized to 60 nm according to the sensitivity results, the optimized device represented in Fig. S7b.

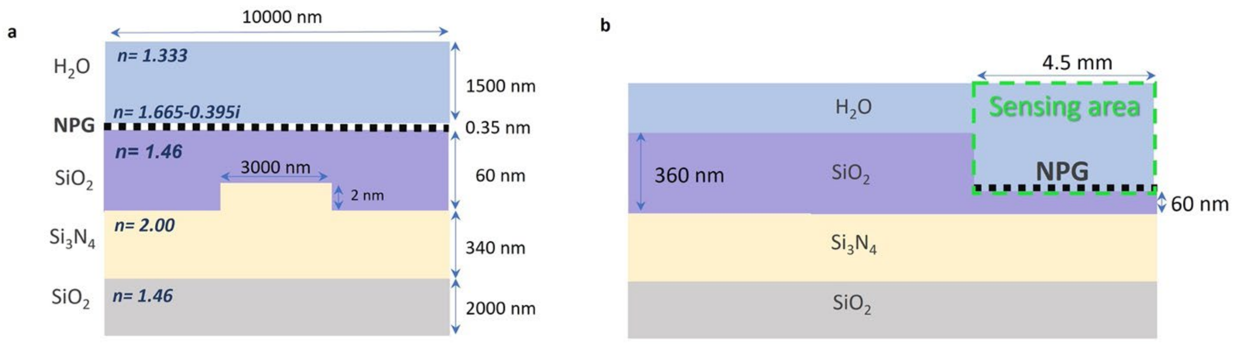

Figure S7. a) Cross section of the optimized NPG-BiMW device including the dimensions and the refractive index parameter of each layer. b) Lateral view of the optimized waveguide, representing the length of the sensing area fully covered with NPG. The schemes represent the bimodal section of the waveguide. Not in scale.

Five DMSO dilutions were prepared with concentration values ranging from 0.37 to 1.23 %. The DMSO refractive index is measured by refractometer (Table 1) and the refractive index variation (Δn) corresponds to the difference between DMSO and milli-Q H_2O refractive indexes.

Table 1. Value of the Δn changes of five different DMSO dilutions prepared. The refractive index of the DMSO solutions and milli-Q H_2O were measured at room temperature.

	Refractive index (n)	**Δn (RIU)**
Milli-Q H_2O (20 °C)	1.33298	
0.37 %	1.33793	0.00495
0.58 %	1.34078	0.00780
0.79 %	1.34360	0.01062
1.01 %	1.34635	0.01337
1.23 %	1.34935	0.01637

To experimentally validate the performance of the SiO_2-BiMW device, the phase shift (ΔΦ) value induced by Δn changes in the sensor surface is extracted from the sensor response (Sr) and plotted in a bulk sensitivity calibration curve to Δn changes. The sensor response (Sr) (Fig. S8a) corresponds to the number of fringes of the sinusoidal signal obtained from the relative intensity variation of the I_{up} and I_{down} measured by a two-section photodetector.

The limit of detection (LOD) value (Fig. S8b) is calculated from the slope of the calibration curve (S_{bulk}) and estimated as three times the standard deviation of the baseline noise (σ_{noise}), expressed as:

$$\mathbf{LOD} = \frac{\Delta\Phi}{\Delta n} = \frac{\Delta S_R}{S_{bulk}} \mathbf{x} \frac{\pi}{2V} = \frac{3\,\sigma_{noise}}{S_{bulk}} \mathbf{x} \frac{\pi}{2V}$$

The factor π/2V translates the Sr variation into a linear signal, and V corresponds to the visibility factor calculated from the amplitude of the Sr fringes pattern.[3]

Calculated the LOD values for the different SiO_2-BiMW devices coated with SiO2 thicknesses of 25, 50, 60, and 100 nm and obtained a variation of the LOD from 4×10^{-6} to 1.4×10^{-5} RIU, with the optimal LOD situated below 10^{-6} RIU (Fig. S8b).

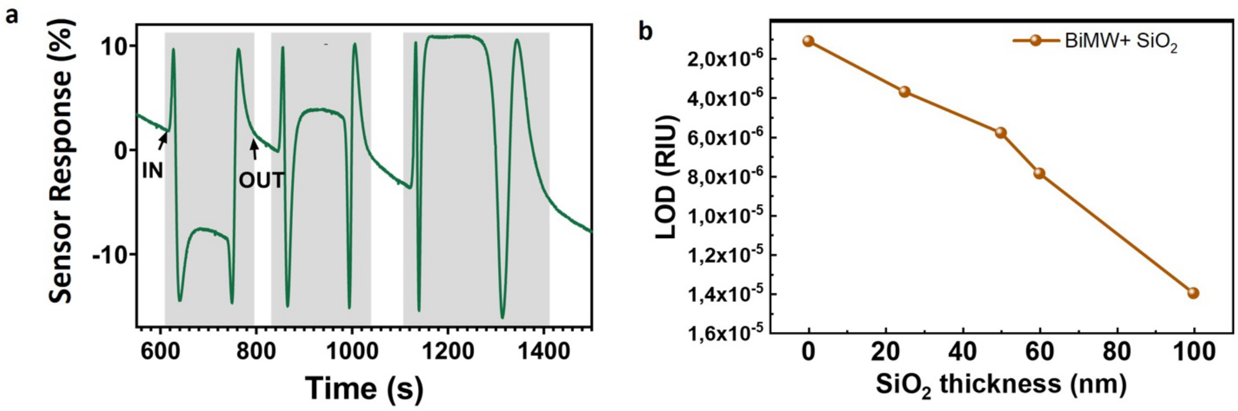

Figure S8. a) Temporal evolution of the Sr for the SiO_2-BiMW device for three DMSO solutions of different concentrations injected. The entrance and exit of the DMSO solution are represented inside the grey area. b) LOD values obtained from the bulk calibration curve for the SiO_2-BiMW devices fabricated with SiO_2 thicknesses of 25, 50, 60, and 100 nm.

E. X-ray photoemission spectroscopy (XPS) characterization

To introduce oxygen-containing functional groups on the NPG surface we employ ex-situ functionalization by oxygen plasma.[5] The reaction is carried out with the sample inserted facing down, separated 1 mm from the support, to reduce the plasma reactivity, described in Fig. S9. The exposition time was evaluated starting at 12 s and increasing to 18 s in different samples. Filled the plasma chamber with O_2 gas for 1 min at a pressure of 445 sccm (Femto, Diener electronic, Germany). Afterward, the samples were annealed for 1 hour at 180 °C on a hot plate.

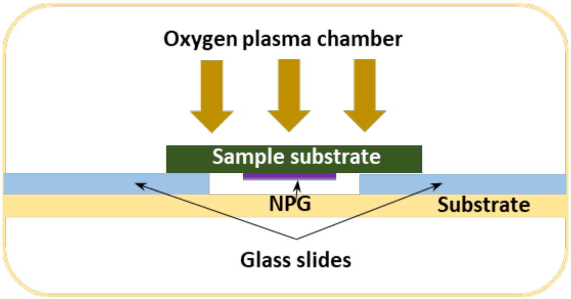

Figure S9. An oxygen plasma treatment is employed to introduce oxygen-functional groups on the NPG surface inside the reactive oxygen plasma chamber. The sample substrate is held facing down by a 1 mm glass slide on the sides.

Characterization of the oxide-NPG sample was performed by XPS and displayed in Figure S10a-b for C 1s and O 1s high-resolution core-level spectra, for different oxygen plasma exposure times. After 12 s of plasma, the area of O 1s increased compared to the pristine sample. In the sample exposed to 18 s of plasma, the C 1s signal showed a high decrease compared to the pristine sample indicating a reduction of the NPG coverage on the surface. Thus, we discarded the 18 s exposure time. The C 1s spectra consist of the C-C peak at 284.4 eV, the C-O at 285.7 eV, the C=O at 286.7 eV, and C=O at 288.1 eV. Furthermore, the shoulders of the O 1s core-level spectra at binding energies 533.5 eV, 532.5 eV, and 531.1 eV are assigned to O-C=O, C=O, and C-O bonds, respectively, evidencing the presence of carboxyl (COOH), epoxy (COC), carbonyl (CHO) and hydroxyl (OH) groups at the surface of the NPG.[5–8]

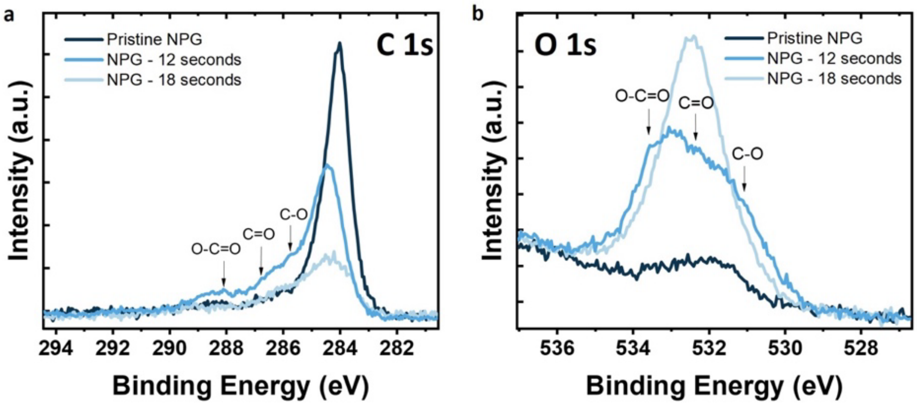

Figure S10. a) C 1s and b) O 1s high-resolution core-level spectra of pristine NPG on Au (111) film under 12 s and 18 s oxygen plasma treatment.

F. Bioassay conditions on the NPG layer

After functionalization of the NPG surface with oxygen plasma, the NPG-BiMW device is mounted on the optical setup to continue with the bioassay on individual waveguides by employing the microfluidics platform. The immobilization of the anti-CRP antibody was monitored in real time (Fig. S11a). Milli-Q water was used as the running buffer during the immobilization step and was then changed to PBS buffer for the detection of C-reactive protein (CRP). Finally, different CRP concentrations ranging from 0.05 to 1 µg/mL, were flowed over the sensor surface and measured the biosensor response in real-time for each analyzed target (Fig. S11b).

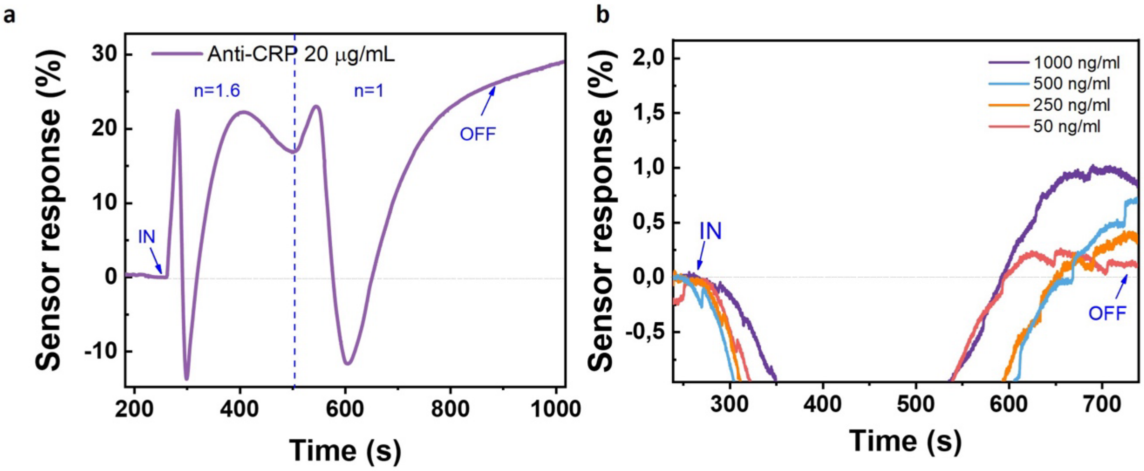

Figure S11. Real-time sensorgrams showing a) the immobilization of 20 µg/mL anti-CRP antibody, and b) the following detection of different CRP protein concentrations (0.05 – 1 µg/mL).

REFERENCES

(1) Okamoto, K. *Fundamentals of Optical Waveguides*; Elsevier, 2021.

(2) Demongodin, P.; El Dirani, H.; Lhuillier, J.; Crochemore, R.; Kemiche, M.; Wood, T.; Callard, S.; Rojo-Romeo, P.; Sciancalepore, C.; Grillet, C.; Monat, C. Ultrafast Saturable Absorption Dynamics in Hybrid Graphene/Si 3 N 4 Waveguides. *APL Photonics* **2019**, *4* (7), 076102. https://doi.org/10.1063/1.5094523.

(3) Dante, S.; Duval, D.; Fariña, D.; González-Guerrero, A. B.; Lechuga, L. M. Linear Readout of Integrated Interferometric Biosensors Using a Periodic Wavelength Modulation. *Laser Photonics Rev.* **2015**, *9* (2), 248–255. https://doi.org/10.1002/lpor.201400216.

(4) Levy, R.; Ruschin, S. Critical Sensitivity in Hetero-Modal Interferometric Sensor Using Spectral Interrogation. *Opt. Express* **2008**, *16* (25), 20516–20521. https://doi.org/10.1364/OE.16.020516.

(5) Zandiatashbar, A.; Lee, G. H.; An, S. J.; Lee, S.; Mathew, N.; Terrones, M.; Hayashi, T.; Picu, C. R.; Hone, J.; Koratkar, N. Effect of Defects on the Intrinsic Strength and Stiffness of Graphene. *Nat. Commun.* **2014**, *5*, 1–9. https://doi.org/10.1038/ncomms4186.

(6) Ma, C.; Xiao, Z.; Puretzky, A. A.; Baddorf, A. P.; Lu, W.; Hong, K.; Bernholc, J.; Li, A. P. Oxidization Stability of Atomically Precise Graphene Nanoribbons. *Phys. Rev. Mater.* **2018**, *2* (1), 1–9. https://doi.org/10.1103/PhysRevMaterials.2.014006.

(7) Rani, J. R.; Lim, J.; Oh, J.; Kim, J.-W.; Shin, H. S.; Kim, J. H.; Lee, S.; Jun, S. C. Epoxy to Carbonyl Group Conversion in Graphene Oxide Thin Films: Effect on Structural and Luminescent Characteristics. *J. Phys. Chem. C* **2012**, *116* (35), 19010–19017. https://doi.org/10.1021/jp3050302.

(8) Cheng, H.-E.; Wang, Y.-Y.; Wu, P.-C.; Huang, C.-H. Preparation of Large-Area Graphene Oxide Sheets with a High Density of Carboxyl Groups Using O2/H2 Low-Damage Plasma. *Surf. Coat. Technol.* **2016**, *303*, 170–175. https://doi.org/10.1016/j.surfcoat.2016.03.028.
